# Supplementary material for: Voices of the vulnerable: Exploring the livelihood strategies, coping mechanisms and their impact on food insecurity, health and access to health care among Syrian refugees in the Beqaa region of Lebanon
Source: PLoS One. 2020 Dec 2;15(12):e0242421. doi: 10.1371/journal.pone.0242421 (PMC7710069; doi:10.1371/journal.pone.0242421)
Supplement: S1 Appendix — (DOCX) [file pone.0242421.s001.docx]

**S1 Appendix. Short Questionnaire with Syrian Refugees**

The following questionnaire will be administered to participants of the focus group prior to the start of the group discussion. The duration of the questionnaire completion should be approximately 15 minutes.

1. When did you arrive to Lebanon (post-2011)?
2. What is the highest educational level you attained?
   1. No formal schooling
   2. Primary education
   3. Middle school
   4. High school/technical diploma
   5. University
3. What is your marital status?

. a. Single

1. Married
2. Divorced
3. Widowed
4. Are you currently employed? If yes, what is the type of your work?
5. Are you the head of your current household? (yes, no)
6. How many household members do you share your household with?
7. How many rooms/separated units are there in your household, (excluding kitchen, bathroom, and balcony, if applicable)?
8. Do you have children, if yes how many? and how many children are under 5 years?
9. Are you currently registered or recorded with the UNHCR? Yes / No
10. How many members of your household are unregistered/unrecorded?

### Food assistance and other humanitarian aid

During the past 3 months have you received any of the following? (Select all that apply) 1- Food packages

1. e-card
2. Conditional cash
3. Other, please specify:

### Household food security (scale)

| **No.** | Question | Response Option |
| --- | --- | --- |
| **1a** | In the past 4 weeks, did you worry that your household would not have enough food? | 1. No 2. Yes |
| **1b** | How often did this happen? | 1. Rarely (1 or 2 times in the past 4 weeks) 2. Sometimes (3 to 10 times in the past 4 weeks) 3. Often (more than 10 times in the past 4 weeks) |

| 2a | In the past 4 weeks, were you or any household member not able to eat the kinds of food you preferred because of a lack of resources? | 1. No 2. Yes |
| --- | --- | --- |
| 2b | How often did this happen? | 1. Rarely (1 or 2 times in the past 4 weeks) 2. Sometimes (3 to 10 times in the past 4 weeks) 3. Often (more than 10 times in the past 4 weeks) |
| 3a | In the past 4 weeks, did you or any household member have to eat a limited variety of food due to a lack of resources? | 1. No 2. Yes |
| 3b | How often did this happen? | 1. Rarely (1 or 2 times in the past 4 weeks) 2. Sometimes (3 to 10 times in the past 4 weeks) 3. Often (more than 10 times in the past 4 weeks) |
| 4a | In the past 4 weeks, did you or any household member have to eat some foods that you really did not want to eat because of a lack of resources to obtain other types of food? | 1. No 2. Yes |
| 4b | How often did this happen? | 1. Rarely (1 or 2 times in the past 4 weeks) 2. Sometimes (3 to 10 times in the past 4 weeks) 3. Often (more than 10 times in the past 4 weeks) |
| 5a | In the past 4 weeks, did you or any household member have to eat a smaller meal than you felt you needed because there was not enough food? | 1. No 2. Yes |
| 5b | How often did this happen? | 1. Rarely (1 or 2 times in the past 4 weeks) 2. Sometimes (3 to 10 times in the past 4 weeks) 3. Often (more than 10 times in the past 4 weeks) |
| 6a | In the past 4 weeks, did you or any household member have to eat fewer meals in a day because there was not enough food? | 1. No 2. Yes |
| 6b | How often did this happen? | 1. Rarely (1 or 2 times in the past 4 weeks) 2. Sometimes (3 to 10 times in the past 4 weeks) 3. Often (more than 10 times in the past 4 weeks) |

| 7a | In the past 4 weeks, was there ever no food to eat of any kind in your household because of lack of resources to get food? | 1. No 2. Yes |
| --- | --- | --- |
| 7b | How often did this happen? | 1. Rarely (1 or 2 times in the past 4 weeks) 2. Sometimes (3 to 10 times in the past 4 weeks) 3. Often (more than 10 times in the past 4 weeks) |
| 8a | In the past 4 weeks, did you or any household member go to sleep at night hungry because there was not enough food? | 1. No 2. Yes |
| 8b | How often did this happen? | 1. Rarely (1 or 2 times in the past 4 weeks) 2. Sometimes (3 to 10 times in the past 4 weeks) 3. Often (more than 10 times in the past 4 weeks) |
| 9a | In the past 4 weeks, did you or any household member go a whole day and night without eating anything because there was not enough food? | 1. No 2. Yes |
| 9b | How often did this happen? | 1. Rarely (1 or 2 times in the past 4 weeks) 2. Sometimes (3 to 10 times in the past 4 weeks) 3. Often (more than 10 times in the past 4 weeks) |

I would like to ask you about all the different foods that your household members have eaten in the last 7 days. Could you please tell me how many days in the past week your household has eaten the following foods?

| Main staples (bread, rice, potatoes) |  |
| --- | --- |
| Pulses (beans and legumes) and nuts |  |
| Fruits |  |
| Vegetables |  |
| Meats and Fish |  |
| Milk, yoghurt, and dairy products |  |
| Sugar (honey, sugar, jams) |  |
| Oil and fat (butter, oil, margarine) |  |
| Condiments (tea, coffee, salt, spices) |  |
