# Supplementary material for: Voices of the vulnerable: Exploring the livelihood strategies, coping mechanisms and their impact on food insecurity, health and access to health care among Syrian refugees in the Beqaa region of Lebanon
Source: PLoS One. 2020 Dec 2;15(12):e0242421. doi: 10.1371/journal.pone.0242421 (PMC7710069; doi:10.1371/journal.pone.0242421)
Supplement: S2 Appendix — (DOCX) [file pone.0242421.s002.docx]

**S2 Appendix. Focus Group Discussion Guide with Syrian Refugees**

| **Session** | **Probing questions** |
| --- | --- |
| **First Session: Open-Ended Story (30 minutes)** | |
| There are a number of labels under which Syrian refugees reside in Lebanon, including registered, recorded, vulnerable, resident under the sponsorship system (economic migrant), or unregistered/unrecorded. In this group, tell us please how do you identify your status? | - Do you mind please telling us how you obtained this status? - Is your status different from those other members of your household? |
| Let’s talk about your life in Lebanon. Being a (registered/unregistered) refugee has bearing on so many aspects of your life.  First, we would like to ask you please to share with us stories about yourself or others from your community who have concerns over their legal status in Lebanon? | - How this affected yours or their lives. - What kind of assistance, if ever, do you get from NGOs and UN because you are a (registered/unregistered) refugee? - How did your/their legal status affect mobility and travel across Lebanon or to Syria? |
| **Food and Nutrition** | |
| We are interested to know about how you secure food for your household? Do you buy your food? | - Do you cover all the expenses? Part of it? - Do you ration your food because you can’t afford it? |
| Do you depend on other sources? | - What are some of these sources? (support from Lebanese or Syrian families? Friends? Debt from food vendors? Food assistance? Others? |
| Do you receive food aid (whether food packages, e-cards, or any form of food assistance)? | - Do you receive these mostly from local or international NGOs, charitable organizations, or religious groups? - And to what extent this access or lack of is affected by your registration status? |
| Do you sometime think that you don’t have enough food on the table? | - Share with us some of your stories on how you deal with food shortage? - Agriculture or cultivation to help you cope with food shortages - Community kitchens, gardens, any other programs or shared facilities that can help you secure food for you and your family? |
| What do you think about the quality or diversity of foods that you and your family members consume? | - Do you or others in your family have access to a variety of foods? What affects your choices? - What about the quality of food you consume? Are you satisfied with it? |
| We are interested to know if children in the house consume food outside of the home? If so, where? |  |
| **Employment and Labour** | |
| We are interested to know the sources of income of Syrian refugees. Do you work for a living? | - Doing what? What about other family members? - How did you manage to secure employment? |
| Is your registration status posing any legal concerns over your employment? | - Is your work considered informal? Legal? - What about the wages, are they affected by your status? |
| Since your arrival to Lebanon, were you ever been unemployed? | - How did you cope without any source of income (selling assets, savings, loans…)? - Did you receive cash assistance or other form of monetary aid? - Is this cash assistance specific to your status or is it given to all refugees alike? |
| Did you learn a new skill to make a living? Did your access to capacity building programs or trainings affected by your legal status? | - What skill (digital/computer, blue color jobs (carpenting, mechanic, plumbing,etc)? Who assisted you to learn these new skills? |
| **Infrastructure and Shelter** | |
| Tell us please about the place where you live now? | Describe it for us. |
| What about access to clean water, electricity and sewage system (each separately). | - Are you having challenges with accessing any of these services, and how are you managing to pay the bills? - Do you have specific entity/ies that helped you secure water, electricity, and other housing-related services? Can you elaborate a bit more about these entities and their services? |
| Do you think your access to these services is similar, better or worse than other refugees in the country ? what about in comparison to the the Lebanese host community? |  |
| **Education** | |
| Do you have any children? Do they go to school? College? | - Is your children’s access to school affected by your family’s status? |
| What are some of the challenges associated with sending your children to schools? | - Are there any specific school policies tagged to your status? |
| If your children are not in schools, are they working? Married? | - Where these decisions affected by your or their legal status? Other factors? |
| **Healthcare** | |
| Describe to us situations where you or any other family member had a health problem, what did you do to seek care? | - Were you able to receive these services? How did you cover the expenses? Did you receive assistance from NGO/UNHCR? If yes, how is this related to your status? - If you were not covered by any assistance, how did you manage to get the service paid? What did you do? |
| Have you or your family members traveled to Syria to obtain healthcare since your arrival in Lebanon? | - What made you do so? |
| **For the focus groups with women only (Women’s Health)** | |
| Have you or someone you know been pregnant in the past year? | - Tell me at what stage of the pregnancy was the first time you visited a center to check on your pregnancy? |
| Can you tell us if you received prenatal health care services and did you face any problems with accessing these services? | - What about after the first visit, were you going for regular visits? Why or why not? - Was there any problem accessing care because of your status? - Did you face any other challenges with the services you were offered? |
| **Family and Social Ties** | |
| Being in another country is very stressful, being a refugee may add to this stress, do you think your situation is affecting your psychology? And if so, how? | - Did your relationship with other family members affected by your legal status (ex. internal conflicts? anger, anxiety?) |
| Since you arrived to Lebanon, have you changed your perspective on the age of marriage or number of children? |  |
| **Wrap Up (10 minutes)**  At the end of the discussions, when the participants have no more points to add summarize the main discussions, and highlight any points that were raised.  Additionally:   - Ask the participants if they have any questions for you.   Tell the group that you are available to speak to any of them in private to answer any personal questions that they may have. | |
