# Supplementary material for: Voices of the vulnerable: Exploring the livelihood strategies, coping mechanisms and their impact on food insecurity, health and access to health care among Syrian refugees in the Beqaa region of Lebanon
Source: PLoS One. 2020 Dec 2;15(12):e0242421. doi: 10.1371/journal.pone.0242421 (PMC7710069; doi:10.1371/journal.pone.0242421)
Supplement: S3 Appendix — (DOCX) [file pone.0242421.s003.docx]

**S3 Appendix. Interview Script with Key Informants.**

Good day and thank you for agreeing to this interview. We appreciate you taking the time to talk to us about your work with refugees and about the experience and challenges you face when providing aid to Syrian refugees based on their legal and registration status.

My name is [Name of interviewer], and assisting me is [name of RA taking notes]. Both of us are from the American University of Beirut, and we would like to ask your approval to tape-record this interview, in order not to miss any important comments. We will not be using any names in the final report, and you can be assured of complete confidentiality. However, if you feel uncomfortable with voice recording the interview please just notify us.

### General

- - Can you please start with introducing the work that your organization is involved in with Syrian Refugees? Area of operation?
  - What is your position/line of work within the organization?

### Field experience

- - Can you describe the population you are serving? Is it based on their legality or registration status?
  - Please tell us if and how the needs of refugees are being assessed (probing: social, health, legal, education), and what are the assessment criteria for providing assistance?
  - What type of services do you provide Syrian refugees with as part of your organization?
  - Do the services your organization provides for refugees differ based on legal or registration status? And if so, how and why?
  - Are there any other labels you use to differentiate service provision, such as vulnerability status, gender, age?
  - What is, in your opinion, the impact of assistance received by Syrian refugees on their livelihoods? (Employment, income, food security, etc.; also probe for impact on their livelihoods, if services were also temporarily interrupted or completely stopped).
  - From your field experience, what do you believe are the biggest challenges faced by unregistered refugees? What are the most unmet needs and required services?
  - What are the main types of coping mechanisms you witnessed among refugees based on their legal or registration status?
  - Do you see these mechanisms sufficient and sustainable? In your opinion, what are some of the consequences of adopting these mechanisms?
  - Any closing remarks or thoughts you would like to share with us re- this topic
