# Supplementary material for: Voices of the vulnerable: Exploring the livelihood strategies, coping mechanisms and their impact on food insecurity, health and access to health care among Syrian refugees in the Beqaa region of Lebanon
Source: PLoS One. 2020 Dec 2;15(12):e0242421. doi: 10.1371/journal.pone.0242421 (PMC7710069; doi:10.1371/journal.pone.0242421)
Supplement: S4 Appendix — (DOCX) [file pone.0242421.s004.docx]

**Appendix S4. Good Reporting of A Mixed Methods Study (GRAMMS) checklist**

| **Guideline** | **Section: page** |
| --- | --- |
| Describe the justification for using a mixed methods approach to the research question | Methods: p. 7, 8 |
| Describe the design in terms of the purpose, priority and sequence of methods | Methods: p. 7 |
| Describe each method in terms of sampling, data collection and analysis | Methods: p. 8-15 |
| Describe where integration has occurred, how it has occurred and who has participated in it | Methods: p. 15 |
| Describe any limitation of one method associated with the present of the other method | Methods: p. 11  Results: p. 27 |
| Describe any insights gained from mixing or integrating methods | Results: p. 27 |

O'Cathain A, Murphy E, Nicholl J. The quality of mixed methods studies in health services research. J Health Serv Res Policy. 2008;13: 92-98
