# Supplementary material for: Voices of the vulnerable: Exploring the livelihood strategies, coping mechanisms and their impact on food insecurity, health and access to health care among Syrian refugees in the Beqaa region of Lebanon
Source: PLoS One. 2020 Dec 2;15(12):e0242421. doi: 10.1371/journal.pone.0242421 (PMC7710069; doi:10.1371/journal.pone.0242421)
Supplement: Attachment — Submitted filename: Response to Reviewers.docx [file pone.0242421.s005.docx]

**October 20, 2020**

**Response to Reviewers**

Dear Editor-in-Chief,

We appreciate the thorough review and the valuable comments that you and the reviewers have provided to our manuscript entitled: “**Voices of the Vulnerable: Exploring the livelihood strategies, coping mechanisms and their impact on food insecurity, health and access to health care among Syrian refugees in Lebanon** ".

In response, we are pleased to submit a revised version of this manuscript, whereby we include below a detailed response to each of the comments and suggestions of the reviewers (*in italics*) followed by the authors’ response. Specific references to alterations in the text of the manuscript are also noted in this letter and were presented in red color within the revised manuscript. All line references in this letter refer to the revised version of the manuscript, as submitted.

Requests from the editors:

1- *Please include additional information regarding the quantitative survey or questionnaire used in the study and ensure that you have provided sufficient details that others could replicate the analyses. For instance, if you developed a questionnaire as part of this study and it is not under a copyright more restrictive than CC-BY, please include a copy, in both the original language and English, as Supporting Information.*

**Authors’ response:** We appreciate the editor’s request. As suggested, additional information was provided in the methods section to further clarify the type of questions included in the short quantitative survey and the methods of analysis. Please see below excerpt from the methods. In addition, a copy of the questionnaire was also provided as supporting information (Appendix S1).

- “The questionnaire (Appendix S1) included sociodemographic information (age, education, casual labor, household conditions), and the Arabic-translated, previously validated version of the Household Food Insecurity Access Scale (HFIAS) [37, 38]. The sociodemographic questions including the HFIAS scale were used in previous studies conducted in Lebanon with Syrian refugees and Lebanese households showing good and reliable results [26, 39, 40]. The HFIAS reflects the food insecurity experience of the participants and their family members in the past four weeks, and it is comprised of nine questions on the occurrence and frequency of food insecurity experience during that period. To calculate the HFIAS score per household, a standard scoring procedure was used whereby 0, 1, 2, 3 points were allocated for “non-occurrence”, “rare”, “sometimes”, and “often”, respectively. The total HFIAS score ranged between 0 and 27, with higher scores indicating higher levels of household food insecurity. Scores were used to categorize households into four levels of food insecurity (food secure, mildly, moderately or severely food insecure) depending on the number of positive responses to questions related to severe conditions, and as per the HFIAS measurement and indicator guide [25]. For purpose of analysis in this paper, the food secure and mildly food insecure households were merged together. In addition, the short questionnaire asked respondents about the frequency of their household’s consumption of eight different food groups over the previous seven days to calculate their food consumption scores (FCS). The FCS is a composite score based on the dietary diversity, food frequency, and relative nutritional importance of different food groups with higher scores referring to higher dietary diversity and frequency of consumption among households [41]. The FCS is later categorized into three groups (poor, borderline and high), as per the standard thresholds [27]. Briefly, the FCS is an index that was developed by the WFP in 1996 and is incorporated in surveys to assess the food security and dietary diversity of vulnerable communities and households [42]. In Lebanon, the FCS represents a key component of the WFP annual surveys conducted to assess the food security status of Syrian refugees [3, 10, 43]. " Pages 11 and 12.

2- *In the Methods, please discuss whether and how the questionnaire was validated and/or pre-tested. If this did not occur, please provide the rationale for not doing so.*

**Authors’ response:** We thank the reviewer for raising this question. The sociodemographic questions in the short questionnaire including the HFIAS scale were used in previous studies conducted in Lebanon with Syrian refugees and Lebanese households showing good and reliable results (Harake, Diab, Kharroubi, Hamadeh, & Jomaa, 2018; Ibrahim, Honein-AbouHaidar, & Jomaa, 2019; Jomaa, Naja, Kharroubi, & Hwalla, 2019) . For the HFIAS (household food insecurity access scale), it was originally developed by the US Agency for International Development-funded Food and Nutrition Technical Assistance Project(Coates, Swindale, & Bilinsky, 2007). This tool was also previously translated to Arabic and validated in Lebanon, showing high internal consistency (Cronbach’s α of 0·91) and reliability (intra-class correlation coefficient of 0·58) (Naja, Hwalla, Fossian, Zebian, & Nasreddine, 2015). As for the food consumption score, this is another index that was developed by the World Food Programme in 1996 and has been used since then in surveys to assess household-level food insecurity and dietary diversity, including the VASyr surveys conducted in Lebanon to assess Syrian refugees’ food security status. Details regarding the validity, prior testing and use of the questionnaire in Lebanon as well as the calculations for both HFIAS and FCS scores were further expanded on in the methods section (see pages 11 and 12).

**References**:

- Ibrahim N, Honein-AbouHaidar G, Jomaa L (2019) Perceived impact of community kitchens on the food security of Syrian refugees and kitchen workers in Lebanon: Qualitative evidence in a displacement context. PLoS ONE 14(1): e0210814. <https://doi.org/10.1371/journal.pone.0210814>.

-Coates J, Swindale A & Bilinsky P (2007) Household Food Insecurity Access Scale (HFIAS) for measurement of food access: indicator guide. <https://www.fantaproject.org/monitoring-and-evaluation/household-food-insecurity-accessscale-hfias>.

-Naja F, Hwalla N, Fossian T et al. (2015) Validity and reliability of the Arabic version of the Household Food Insecurity Access Scale in rural Lebanon. Public Health Nutr 18, 251–258.

- El Harake, M.D.; Kharroubi, S.; Hamadeh, S.K.; Jomaa, L.(2018). Impact of a Pilot School-Based Nutrition Intervention on Dietary Knowledge, Attitudes, Behavior and Nutritional Status of Syrian Refugee Children in the Bekaa, Lebanon. Nutrients 10(7), 913; <https://doi.org/10.3390/nu10070913>.

-Jomaa, L., Naja, F., Kharroubi, S., & Hwalla, N. (2019). Prevalence and correlates of food insecurity among Lebanese households with children aged 4-18 years: findings from a national cross-sectional study. Public health nutrition, 22(2), 202–211. <https://doi.org/10.1017/S1368980018003245>.

3- *We note that you have indicated that data from this study are available upon request. PLOS only allows data to be available upon request if there are legal or ethical restrictions on sharing data publicly. For more information on unacceptable data access restrictions, please see* [*http://journals.plos.org/plosone/s/data-availability#loc-unacceptable-data-access-restrictions*](http://journals.plos.org/plosone/s/data-availability#loc-unacceptable-data-access-restrictions)*.*

**Authors’ response:** The editors and reviewers’ requests are well-received. Kindly note that copies of the questionnaire, focus group script and data analysis document are all submitted as supplementary material to the manuscript. With regards to the availability of data, kindly note that we have ethical restrictions that we would like to highlight here:

- Please note that the language written in the oral consent forms and the focus group discussion script assured participants at the beginning of the study that their data will remain confidential: *“Your individual privacy and the confidentiality of the information you provide will be maintained in all published and written data analyses resulting from the study. Only the research team will have access to the questionnaires, recordings and transcripts from these interviews that will be safeguarded, stored on a password-protected computer at the American University of Beirut (AUB), and not disclosed to anyone.”*
- The issue was also discussed with the AUB Institutional Review Board, and we were advised not to proceed in sharing the data using the online data repositories, but rather to include the statement below, as also submitted on the system upon submission:
- “Data cannot be shared publicly because of confidentiality and privacy concerns. The American University of Beirut Institutional Review Board (IRB) approved the study, and the consent document the IRB approved assured participants that their data would not be shared beyond the research team and as aggregated in publications. The de-identified data for the study may be made available to investigators who contact AUB in accordance with institutional policies. Please note that AUB policies require AUB investigators to retain custody of research data, unless Non-Disclosure Agreements (NDA) have been signed prospectively with investigators/collaborators in other institutions. You can also contact AUB IRB office irb@aub.edu.lb for any additional inquiries related to human subjects' data for research purposes.”

As noted, the de-identified data for the study may be made available to investigators who contact AUB and follow the institutional policies regarding developing and signing NDA agreements to allow interested investigators to access this data.

**Reviewer #1:**

*1. The study provides a relevant and useful analysis of the situation and needs of an often hidden population which have not been considered sufficiently in the humanitarian response, and as such provides useful data for the agencies and government. It is a pity that it has taken quite a long time to publish the findings, however the report will still be useful I am sure.*

**Authors’ response:** We thank the reviewer for finding the study findings to be relevance and suitable to address a hidden population that has not been sufficiently addressed in previous studies on humanitarian crises and responses. We look forward to sharing the study findings and implications through the PLOS One in a timely manner, after unfortunate delays in the revision process of the manuscript in another peer-reviewed journal.

*2. As the study looked only at the Beqaa valley which the report states represents just over a third of the refugees in Lebanon, the title and conclusions may be more accurate if they were changed to reflect this geographical coverage, rather than all of the Syrian refugees in all of Lebanon.*

**Authors’ response:** Thank you for the suggestion. The title and conclusion have been amended to specify the geographical location of the study and its conclusions as reflective of Syrian refugees in the Beqaa region.

- “In conclusion, the study findings highlight the high dependency of Syrian refugees in the Beqaa region on formal assistance, when received based on their registration status, albeit being insufficient.” Line 733, page 36.

*3. Was there any instance in which data triangulation efforts revealed discrepancies, and if so how were these handled?*

**Authors’ response:** Our study triangulated qualitative data from focus group discussions (FGD) with in-depth interviews. The data triangulation from the FGDs and in-depth interviews provided a complimentary perspective on the findings, thus increasing the study’s credibility. Following the synthesis of FGD results, findings from the interviews were analysed using the same identified themes and subthemes and presented in a narrative form. Within each theme, findings from both FGD and in-depth interviews were presented to highlight any discrepancies. For example, under “Informal employment and exploitative work conditions” subtheme, only key informants reported that refugees resort to sex work and begging. This discrepancy was highlighted in the results section (p.18) and the discussion. . Please see excerpts from the manuscript:

- Results: “). Some key informants reported that Syrian women resort to socially less acceptable jobs, such as sex work and begging: “So it is more of a coping mechanism for women who are willingly doing that, but for younger girls it is sometimes a coping mechanism and sometimes exploitation against their will” (Local NGO - Int. 9). Nevertheless, sex work and begging were not reported by refugees during the FGD.” Line 372, Page 20.
- Discussion: “According to Bartels et al (2018), in many instances, Syrian refugee girls are ready to accept the proposals from well-settled men to secure more protection and escape the hardship conditions of their families [57]. The dire living conditions, poverty and insecurity were also reported in other studies as key motives for the households to take that decision on the behalf of their daughters and marry them off at early age [51, 54]. Sex work is another informal coping mechanism that was adopted by Syrian refugees to overcome their economic hardships, yet it was reported only by the key informants in the present study. Given the sensitive nature of this practice and the associated cultural, religious and legal taboos of sex work [3, 52, 53], it was not surprising to observe a discrepancy between Syrian refugees and key informants on the reporting of sex work as a form of informal employment. Nevertheless, these findings highlight the need for further exploration of sex work as a form of informal employment among this vulnerable population.” Line 586, Page 29.

Furthermore, data was further triangulated by employing a mixed method study. Qualitative data was supplemented with quantitative data from the questionnaire, and the findings were analysed for concordance and discordance, which are reported under the integrated analysis section (Page 27).

*4. Is it possible that the much higher number of female participants (82 women, 40 men) influenced the findings in any way?*

**Authors’ response:** Thank you for your question. We acknowledge that there was a higher number of female respondents (n = 82) to male respondents (n = 40) completing the quantitative survey at the beginning of the FGDs, nevertheless it is worth noting that the total number of FGDs conducted with both genders were on average balanced (FGD with women = 10, FGD with men = 9, please see page 8). The lower number of men per FGD can be explained by the lower availability of men during the regular work days to take part in the study compared to women who were more likely to be present at home for child care and other related activities. Kindly see the limitations section slightly updated to reflect this issue:

- “Low response rate on the short-questionnaire was also noted among the men and unregistered refugees. This could be explained by time constraints and the fear of disclosing sensitive information that may risk respondents to be exposed to the authorities. To overcome these challenges, study participants were assured of the confidentiality and privacy of the data. In addition, the research team members received extensive trainings at the beginning and throughout the data collection period given the sensitive nature of the discussions and the legality concerns of the interviewed participants.” Line 728 Page 35.

We would like to also highlight that the gender balance was considered throughout the different phases of the study from recruitment to data collection, analyses and interpretation. Please see the comment below regarding gender balance in the reporting of the study findings to further elaborate on this point.

*5. It would be useful to have a copy editor review the document, check for a few instances of unfinished sentences and occasional spelling mistakes (including the correct full name of UNHCR).*

**Authors’ response:** We appreciate the reviewer’s comment. The manuscript was revised to ensure that grammatical mistakes and incomplete sentences are revised as needed. In addition, abbreviations (ex. UNHCR) were also fully spelled out when first introduced in the manuscript.

*6. Would be useful to mention the gender balance of those interviewed and any gendered implications of the findings.*

**Authors’ response:** Thank you for this insightful comment. The role of gender in our study was considered from its inception. Gender dynamics influence access to healthcare, livelihood strategies and coping mechanisms. As such, we divided the FGDs based on gender in order to understand gender-specific findings and saturation was assessed separately for both groups. In our analysis, we included findings based on themes that appeared in FGDs that were conducted with women and men to ensure that the findings have a gender balanced narrative. There were a number of gender-specific findings that were highlighted in our results, such as (1) informal employment in house cleaning and sex work reported by key informants with regards to female Syrian refugees (p. 20), and (2) prioritization of medical conditions for which to seek care was reported only by women and not men (p. 25).

*7. Would be useful to give a little more explanation of how early marriage is considered an informal livelihood strategy.*

**Authors’ response:** Thank you for raising this important question. In the present study, both men and women respondents expressed early marriage for girls as a culturally acceptable practice and a way to overcome their financial burdens. Similarly, early marriage has been reported in other research studies and reports from non-governmental entities working with Syrian refugees in Lebanon as a form of survival and security. According to a report published by Abaad and Queens University exploring child marriage among Syrian refugees in Lebanon, Syrian girls were found to choose early marriage as a way out of their unfavorable living conditions (Saja et al 2018). Similarly, other reviews and studies echoed financial needs and economic concerns as key motives for early marriage, as girls would either choose this option themselves out of desperation given their limited schooling and low-skill employment opportunities or their families would marry off their daughters at an early age to help reduce the overall financial burden (El Arab and Sagbakken 2019, Bartels et al 2018, Mourtada et al 2017). The discussion section was revised to provide further explanation as to how early marriage was considered an informal livelihood strategy for Syrian refugee families (page 29).

References:

- Michael Saja, Roupetz Sophie, and Bartels Susan. Caught in Contradiction: Making Sense of Child Marriage Among Syrian Refugees in Lebanon. ABAAD Resource Centre for Gender Equality. May 2018.

- R. El Arab & M. Sagbakken (2019) Child marriage of female Syrian refugees in Jordan and Lebanon: a literature review, Global Health Action, 12:1, DOI: 10.1080/16549716.2019.1585709.

- Bartels SA, Michael S, Roupetz S, et al. Making sense of child, early and forced marriage among Syrian refugee girls: a mixed methods study in Lebanon. BMJ Glob Health. 2018 Jan 1;3:e000509.

- Mourtada R, Schlecht J, DeJong J. A qualitative study exploring child marriage practices among Syrian conflict-affected populations in Lebanon. Confl Health. 2017 Nov;11:27.

*8. Reasons for data not being available are not provided. If the data sets are anonymized in the way described, are there still confidentiality issues?*

**Authors’ response:** We thank the reviewer for raising this concern. Though data is anonymized, the oral consent form and the focus group discussion script assured participants that their data will remain confidential and will not be shared outside the research team. (“Your individual privacy and the confidentiality of the information you provide will be maintained in all published and written data analyses resulting from the study. Only the research team will have access to the questionnaires, recordings and transcripts from these interviews that will be safeguarded, stored on a password-protected computer at the American University of Beirut (AUB), and not disclosed to anyone.”). This issue was also discussed with the AUB Institutional Review Board, and we were advised to provide the de-identified data to investigators who contact AUB and follow the institutional policies regarding developing and signing NDA agreements with the primary investigators.

*9. Not sure of the relevance of the p-values here? What do they compare?
Interesting to note the big discrepancy in scores between HFIAS and FCS, particularly amongst those not receiving assistance. Did data triangulation / observation reveal anything about why it would be that food consumption was not so bad, while HFIAS scores were very bad?*

**Authors’ response:** We appreciate the reviewer’s comment. The p-values as presented in Table 2 refer to significant differences in the sociodemographic characteristics between the three groups of Syrian refugees included in the study, namely the registered refugees with assistance, those registered not receiving (without) assistance, and the unregistered/unrecorded group of refugees without assistance. For example, significant differences were noted between the three groups of refugees based on registration and assistance on several characteristics including years of stay in Lebanon, number of children, crowding index, household food insecurity and food consumption. A summary of these discrepancies was noted in the results and was included as part of the discussion when referring to the challenges faced by Syrian refugees and the severity of these challenges being most pronounced among the unregistered refugees who do not receive formal assistance. The exact p-values were removed from the table and were instead replaced with the symbols (*, **) and a footnote was added to refer to the significance levels.

As for the discrepancy between HFIAS scores (levels of food insecurity) and the FCS among the refugee groups in the present study, this can be explained by the different dimensions that are being captured by each of these indicators. For example, the HFIAS indicator helps in assessing the food insecurity experience of the participants and their family members in the past four weeks through asking nine questions about the occurrence and experience of participants in terms of limiting the quality (kinds and variety of food eaten) as well as limiting the overall quantity of food consumed. The HFIAS score, as with other food insecurity experience scales, is intended to measure the food insecurity experience from the perspective of people themselves. On the other hand, the food consumption score (FCS) indicator captures information about usual household diet, as it incorporates consumption frequency over a seven-day period. FCS includes questions about specific food groups consumed or not consumed by household members during the past week, and is used a proxy for the dietary diversity and energy sufficiency (quantity dimension), rather than the experience and fear of being food insecure which is captured in the former HFIAS indicator covering both the quality and quantity dimensions. It is worth noting that similar discrepancies were also noted in the vulnerability assessment of Syrian refugees in Lebanon (VASyR 2019) between the proportion of Syrian households that were considered as food insecure (92%), and the proportion of households with poor or borderline food consumption (25%).

In summary, within our study, we found that the majority of refugees without assistance (registered or unregistered) were categorized as moderately or severely food insecure and at least two-thirds had poor or borderline food consumption scores compared to the refugees receiving assistance. These results further highlighted the differences in the food insecurity experience and quantity/energy sufficiency between those who rely on assistance and those who do not using different food insecurity and dietary diversity indicators. Please see the results section (page 15) highlighting the significant differences between Syrian refugees receiving assistance and those not receiving assistance on both the HFIAS and the FCS scores.

*10. On the issue of debt, was it considered that being able to access debt is not always negative, but a sign that the people/institutions providing credit have some confidence in getting repaid and therefore a sign of some capacity to pay by the one taking the loans? Otherwise they would not keep increasing credit. (p.28)*

**Authors’ response:** Thank you for your comment. Both reliance on debt and social exchange were considered common strategies adopted by the refugees to overcome their economic challenges and constraints. With respect to the accumulation of debt by refugees, it was mostly from shopkeepers and pharmacists, whose livelihood in turn was dependent on the assistance that refugees received allowing the latter to repay their loans. Other sources of debt included the ‘shaweesh’, who was often employing the refugees and renting the tents/land, thus, he could leverage these loans. Refugees reported that they tried to repay their debts at the end of each month, but in most cases they could not pay them in full. The accumulation of debt was also emphasized by key informants as a negative strategy since it led some refugee families to accumulate high amounts of debt which forced them to rely on child labor and other negative coping strategies to repay their loans. On the other hand, the social exchange between refugees and their neighbors, who were tied by social bonds and cultural factors, were perceived more positively. Social exchange was particularly evident among the unregistered refugees who were not entitled to formal assistance, yet were able to share food and assistance with their family members, friends and neighbors receiving assistance. Our results with regards to the accumulation of debt as a worrisome livelihood strategy was also reflected in the VASyr 2019 survey, which showed that 9 out of 10 households were in debt and that the two main sources of income for the registered refugees were WFP assistance and informal debt from friends and shops. These findings further highlight the challenges that refugees face to cover necessities and that may become hard to break the cycle of poverty. To further clarify the differences in practices and perceptions of refugees towards debt and social exchange, we made few edits to the manuscript text as follows:

- Results (p. 21 and 22): “Dependence on debt was also echoed by key informants as a negative livelihood strategy and coping mechanism, particularly amongst refugees who were unregistered: “It is true that debt is common regardless of registration status, but for the others (referring to those receiving assistance) every month they pay back some of their debt, the problem is for those unregistered with the UNHCR or registered and not benefitting from assistance, their debt is very big, large, very, and this affects a lot of things such as sending their children to work, sending the wives for extra work, the type of work becomes more difficult because the number of unregistered refugees is increasing, and as such work opportunities are decreasing so they go to work on the roads in prostitution for example and other jobs. The debt is a big problem because all Syrians are now in debt” (Local NGO - Int. 9). “Line 411.Page 21.

In addition to accumulation of debt, unregistered refugees expressed reliance on more positive mechanisms such as social exchange within Syrian refugee families and among friends and neighbors to share food and/or assistance…”

- Discussion (p. 29): “Accumulating debts was another common negative livelihood strategy reported by our study participants adding to the refugees’ vulnerability and compromising their health. Refugees not receiving assistance reported that they continued to accumulate debt, whereas their refugee counterparts who were registered with UNHCR and receiving assistance were able to cover some of their debt at the end of each month. In line with our study findings, 9 out of 10 registered Syrian refugee households in the VASyR were in debt, and the main reasons for borrowing money were for food, rent and health care payments, which shows that even registered refugees continue to lack resources to cover their essential needs[10]. While debt is a formal strategy meant to maintain normalcy and dignity by increasing the purchasing power for essential items, such as nutritious food, i.e. fruits and vegetables, or securing a shelter; it compromises the psychological status of individuals by adding another layer of complexity and stress to the fragile status of refugees [58]. Social exchange of assistance, goods and services, on the other hand, was noted as an acceptable livelihood strategy among the Syrian refugee community within their respective ITS.” Line 597,Page 30.

**Reviewer #2:**

*1. Suggestion: take out the details of the methodology from the abstract (leave the highlights only)*

**Authors’ response:** Thank you. We have shortened the methodology section in the abstract leaving the highlights, as suggested.

*2. Line 55: sentence incomplete*

**Authors’ response:** Thank you. The sentence has been completed as follows:

- “Of note, as of May 2015, and in response to the GoL decision, UNHCR suspended registrations of Syrian refugees.” Line 55, Page 4.

*3. It would also help in the intro to contextualize the case of Lebanon as a refugee host country (especially historically and considering the disproportionality of refugee v citizens)*

**Authors’ response:** We thank the reviewer for this suggestion. The introduction was revised to contextualize the case of Lebanon as a host country preceding the Syrian refugee crisis (page 4).

- “Lebanon, a small middle-income country of six million people, has the largest concentration of refugees per capita and the fourth largest refugee population in the world [2-4]. Historically, Lebanon has been a host country for refugees who have fled wars and persecution in their home countries, including refugees from Palestine with the 1948 Palestinian exodus, from Iraq post the Persian-Gulf war in the 1990s and the 2003 invasion, as well as forcibly displaced individuals from Syria with the onset of the war in 2011[5-7].”Line 46, Page 4.

*4. Line 66 suggestion to define the meaning and stats of registered v unregistered refugees*

**Authors’ response:** Thank you for the suggestion. A brief definition and description of the registered, recorded and unregistered individuals was added to the introduction section.

- “Of note, as of May 2015, and in response to the GoL decision, UNHCR suspended registrations of Syrian refugees. In the meantime, UNHCR has been “recording” rather than “registering” individual refugees where basic personal data of newcomers are being recorded, taking into consideration protection and assistance. In parallel, an unidentified number of refugees have never been registered nor recorded and are referred to as ‘unregistered’.” Line 55, Page 4.

*5. Line 74: what about the xenophobic and anti-refugee sentiments and discrimination that are increasingly reported in Lebanon specifically.*

**Authors’ response:** We thank the reviewer for pointing this issue. The introduction section was revised to indicate the increased social tension between refugees and host communities, and the increased sentiments of discrimination and intolerance experienced by refugees due to competition over limited resources and livelihood opportunities.

- “Syrian refugees also continue to face heighted social tension with the host community due to competition over limited livelihood and job opportunities in a resource-strained, small country like Lebanon [17, 18]. These conditions have further exacerbated the living conditions of refugees and exposed them to different forms of discrimination, prejudice and scapegoating by the Lebanese host communities [17, 18].” Line 78, Page 5.

*6.Line 80: citation for definition of coping? might benefit from grounding coping mechanisms in previous literature defining and discussing the concept in different other contexts. E.g. (Camino et al, (2005). Szczepanikova (2005), Franz (2003), Säävälä (2005).*

**Authors’ response:** Thank you. The intro section was revised to clarify the definition of coping as used in the study, while also grounding it in the previously published literature on coping mechanisms adopted by refugees and displaced individuals in stressful situations. Please see revised excerpt:

- “Coping mechanisms are the actions performed by individuals and households to solve any issue that they perceive as problematic[22], and the scientific literature is rich with examples of different coping mechanisms and strategies adopted by displaced individuals and refugees under stressful situations [23]. Some of these coping strategies focus on the emotional wellbeing of affected individuals, such as seeking social support and using religion and faith to overcome negative feelings and emotions[24, 25] , while other coping mechanisms adopted by refugees include food and non-food related mechanisms to overcome food shortages and economic constraints [26-28]. ” Line 89, Page 6.

*7. It might help at the end of the intro to map the study (e.g. in the next section we will discuss x, then y, then z and will conclude with a,b,c).. also, how such study can benefit us and who would it benefit?*

**Authors’ response:** As suggested, the last paragraph in the intro section provided a brief presentation of the subsequent sections including the methods adopted in the present study to meet the research aim and objectives as well as the implications of the study findings and whom it may benefit. Please see pages 6 and 7.

- “Thus, an in-depth understanding of the livelihoods and coping mechanisms adopted by all Syrian refugees and a comparison by registration status are much needed. This study aimed to explore the livelihoods, coping strategies and access to health care among Syrian refugees in the Beqaa region in Lebanon based on their registration status (registered vs. unregistered) and accompanying formal assistance. In this paper, a mixed methods approach with more emphasis on the qualitative design was adopted to address the research objectives. Further details were provided in the subsequent section. Findings from the present study can assist humanitarian workers, public health professionals, and decision makers in improving their understanding of the livelihood strategies and challenges faced by Syrian refugees in Lebanon, particularly the ‘invisible’ group of unregistered and unrecorded refugees. In addition, these findings can help policy makers and practitioners in developing new programs and services that are sensitive to the needs of hard-to-reach groups of refugees to improve their living conditions and their access to health care and other basic services.” Line 107, Page6.

*8.Strong methods section – only comment is around the translation: it would be helpful to reflect a bit on any challenges or loss of meaning during this process.*

**Authors’ response:** Thank you. In order to avoid losing meaning during translation, the research team decided to not translate the transcripts prior to thematic analysis (line 218, p. 10). As all members of the research team are fluent in Arabic the data was analyzed in Arabic and only the quotes that were used in the manuscript were translated to English. Translation was not conducted verbatim, but taking colloquial terms and inflections into consideration and translating while keeping the quote as close in meaning to its original as possible. This issue was further clarified in the methods section as such:

- *“*Audio recordings were transcribed verbatim concurrently with the data collection. The research team avoided translation while transcribing to observe fidelity of meanings [45]. Instead, data were analyzed in Arabic, and only the quotes that were used in the manuscript were translated from colloquial Arabic to English.” Line 253, Page 13.

*9. Any explanation of the disproportion between men and women in the sample?*

**Authors’ response:** Thank you for raising this question. First, we would like to acknowledge that although there was a higher number of female respondents (n = 82) to male respondents (n = 40) completing the quantitative survey at the beginning of the FGDs, the total number of FGDs conducted with both genders were on average balanced (FGD with women = 10, FGD with men = 9, please see page 8). The lower number of men per FGD can be explained by the lower availability of men during the regular workdays to take part in the study compared to women who were more likely to be present at home for childcare and other related activities. In addition, we noted faster saturation with men who were less interested to extensively discuss some of the study topics, especially food security and healthcare, which are often seen as part of the women’s role in the family.

Kindly see the limitations section slightly updated to reflect this issue:

- “Low response rate on the short-questionnaire was also noted among the men and unregistered refugees. This could be explained by time constraints and the fear of disclosing sensitive information that may risk respondents to be exposed to the authorities. To overcome these challenges, study participants were assured of the confidentiality and privacy of the data. In addition, the research team members received extensive trainings at the beginning and throughout the data collection period given the sensitive nature of the discussions and the legality concerns of the interviewed participants.” Line 728, Page 35.

We would like to also highlight that the gender balance was considered throughout the different phases of the study from recruitment to data collection, analyses and interpretation. We conducted separate FGDs based on gender in order to understand gender-specific findings and to respect the cultural preference for distinct FGD due to the sensitivity of certain topics that may arise during the discussions. In addition, saturation of data was also assessed separately for both groups (men and women). These issues were reflected in the methods section , as per the below:

- Methods: “Refugees were purposively selected to be 18 years and older, residing in Lebanon for more than 3 months, and belonged to one of the three categories below (each further divided into groups of men and women): 1- refugees registered or recorded with UNCHR receiving formal assistance and healthcare subsidies (4 FGDs with women and 3 FGD with men), 2- refugees registered or recorded with UNHCR but not receiving any assistance for more than 6 months (3 FGDs with women, 2 FGDs and 1 structured interview with men), and 3- unregistered and unrecorded refugees not eligible for assistance or healthcare subsidies (3 FGDs with women and 4 FGD with men).” Line 151, Page 8.
- “For cultural appropriateness, gender congruence was observed, i.e. men moderators facilitated the men’s FGDs and women moderators for women’s groups.” Line 230, Page 12.
- “The decision to conclude data collection was done once we reached data saturation by group (registered with assistance, registered non-assistance, and unrecorded) and by gender within each group.” Line 237, Page 12.

In our analysis, we also included findings based on themes that appeared in FGDs that were conducted with women and men to ensure that the findings have a gender balanced narrative. There were a number of gender-specific findings that were highlighted in our results, such as (1) informal employment in house cleaning and sex work reported by key informants with regards to female Syrian refugees (page 20), and (2) prioritization of medical conditions for which to seek care was reported only by women and not men (page 25).

*10. Apologies if I missed that part: is there a clear distinction in the analysis, in addition to registered and unregistered refugees, between urban refugees (in the city or even rural areas) and those in camps? I think it is an important distinction that might be helpful to highlight more clearly throughout the emerging themes section (it also has an important empirical contribution to understanding the experiences of refugees in encampment v those in the city)*

**Authors’ response:** We appreciate the reviewer’s comment. Kindly note that our study population only consisted of refugees residing in informal tented settlements (ITS). As per the Government of Lebanon policy, no formal refugee camps were established in Lebanon in response to the influx of Syrian refugees (UNHCR). Refugees have been residing in either residential shelters (ex. apartment/concierge room in a building), non-residential shelters (ex. factory, farm, warehouse), or non-permanent structures (ex. ITS) with a noticeable shift away from residential shelters since 2017. The majority of the non-permanent structures/ITSs were located in Baalbek-El Hermel, Beqaa and Akkar regions. Given that our study was conducted in the Baalbek and Hermel governorate (North of Beqaa), where the majority of refugees reside in ITSs, we recruited refugees residing in these informal settlements.

Another important issue to highlight is that the present study aimed to recruit Syrian refugees who were unregistered and unrecorded with the UNHCR in Lebanon. This group of refugees represent a particularly ‘hard to reach’ group, who are not part of any government or UN roster and lack a legal status in the country. In fact, previous reports have stated that the unregistered refugees may be avoiding registration with UNHCR and other humanitarian or governmental agencies for fear of arrests and deportation (Project, 2013). It was also noted that poorer refugees and those living in non-permanent shelters (ITS) were also less likely to hold legal residency permits (WFP survey 2019). Taking into consideration these factors and after consulting with our field partners, namely Action Against Hunger, who assisted the team in identifying areas in the Beqaa region to reach this ‘hidden’ group, we considered the ITS as the adequate setting for recruiting the refugee participants.

*11. Line 340: child labor normalized where? In Syria? Lebanon? Due to displacement?*

**Authors’ response:** Thank you for the question. The question asked during the FGD and key informant interviews focused on the current situation. Child labor was reported by both refugees and key informant interviews as common in Lebanon as a result of their displacement. We modified the sentence to reflect our findings and highlight that our results are reflective of the situation in Lebanon.

- “According to key informants, child labor is widespread and normalized in Lebanon, further increasing rates of children who were out of school.” Line 380, Page 20.

*12. Line 350: same thought: child marriage is acceptable among the refugees only? Or did it exist prior to displacement? Did it increase after? Also, a definition is needed for these thorny notions. And what’s the age threshold?*

**Authors’ response:** Thank you for the question. Early and child marriage was present in Syria prior to displacement, however, a few studies indicated an increase since displacement in Lebanon(DeJong et al., 2017; Mourtada, Schlecht, & DeJong, 2017) .

In our findings we focused on the current situation of Syrian refugees in Lebanon, whereby both refugees and key informants reported that early marriage was normal and common during their displacement Lebanon. The definition of early marriage is any marriage between a child under the age of 18 and another adult or a child. The majority of respondents were aware of the concept when asked the question and we did not provide a formal definition except in the cases where the participant asked for a clarification(UNICEF, 2020).

**References**

- Mourtada R, Schlecht J, DeJong J. A qualitative study exploring child marriage practices among Syrian conflict-affected populations in Lebanon. Confl Health. 2017 Nov;11:27.
- DeJong J, Ghattas H, Bashour H, Mourtada R, Akik C, Reese-Masterson A. Reproductive, maternal, neonatal and child health in conflict: a case study on Syria using Countdown indicators. BMJ global health. 2017;2(3):e000302

*13. Line 377: this is very interesting and important and might benefit from being highlighted and grounded a bit more in literature on refugee resilience and coping.*

**Authors’ response:** Thank you for your comment. Both reliance on debt and social exchange were considered common strategies adopted by the refugees to overcome their economic challenges and constraints. Few revisions were done in the results (pages 21 and 22) and discussion section (page 30) to further highlight how the accumulation of debt was considered a common negative livelihood strategy adding to the vulnerability of the refugees, particularly those who were not receiving UN (formal) assistance. On the other hand, the social exchange of goods and services was considered a more acceptable livelihood strategy among the Syrian refugees, which reflects an increased resilience among the refugee community.

*14. Line 444: this is a good example to identify any distinction between those in camps v those in the city.*

**Authors’ response:** Thank you for your comment. Unfortunately our sample and target population only included refugees residing in informal tented settlements (ITS) in the Beqaa valley. As such, we are unable to make comparisons between urban and rural refugee populations in our results.

It is also worth highlighting that in contrary to Jordan and Turkey among other neighboring countries, Lebanon has no official ‘camps’ for Syrian refugees. Instead, the majority of Syrian refugees reside in overcrowded non-permanent shelters and ITS. Our study aimed to include registered and unregistered refugees, the latter are considered a particularly hard to reach group that lacks official status in Lebanon and is likely to reside in informal settlements within rural areas. This represents one of the justifications for the choice of location for conducting the study in the Beqaa region that is close to the Syrian borders and that is believed to have a large, though still unknown, number of unregistered refugees. Please see the methods section providing further explanation for the study setting (page 7).

*15. Line 519: what is this assessment? What is its source and what makes it reliable? Also, any citation?*

**Authors’ response:** Thank you for the comment. We have added a reference of the VASyR assessment that we discuss in the subsequent paragraph. The Vulnerability Assessment of Syrian Refugees in Lebanon (VaSYR) is an annual assessment conducted by UNHCR, UNICEF and WFP (the leading international humanitarian agencies providing services to Syrian refugees in Lebanon). The VASyR helps in assessing the food security status, vulnerability and the access to basic services among a representative sample of registered Syrian refugees, utilizing a multi-sectoral approach. Our study findings were also compared and discussed in light of some of the results highlighted in VASyR in the discussion section.

*16. Line 540-543: very important reflection>> the gap between what’s told and what’s experienced.*

**Authors’ response:** Thank you. We agree that the discrepancy observed between what is being told by the key informants (who are representatives from local and international humanitarian agencies and organizations working directly with Syrian refugees in the study area) and what is being experienced by refugees themselves is very important to highlight. For a culturally and socially unacceptable practice such as sex work that can also have legal implications in Lebanon, the authors were not surprised to note this discrepancy. However, our findings allude to the need for further exploration of prostitution as an irreversible coping mechanism that can have serious implications on the health, safety and security of the affected refugees and their families. The discussion section was revised to reflect these observations (pages 21 and 22).

*17. I’m interested in the definition of vulnerability, specifically how this study defines it.*

**Authors’ response:** We appreciate the question raised by the reviewer. Indeed, vulnerability is a widely used concept in academic research and social work. The definitions for vulnerability do vary between studies and have been contested to be unclear, and in certain instances the term vulnerability was associated with stigmatization of individuals or groups who are less fortunate or in need (Virokannas et al 2018). Albeit the importance of this growing literature and the need for novel and more analytical tools to be used when defining vulnerability, in the present study, we define vulnerability as a “function of how a household’s livelihood would be affected by a specific hazard (ex. crisis, emergency) and how it would manage to cope with this impact”. This definition relies on the vulnerability assessment and mapping done by international agencies such as the World Food Programme; and it is along the lines of the DFID sustainable livelihoods framework that considers that vulnerability “can emerge when human beings have to face harmful threat or shock with inadequate capacity to respond effectively”.

To further clarify this issue, a statement was added to the introduction section to highlight how the authors are defining vulnerability in this particular study (see page 6).

- “The term vulnerability, as defined in the present study, refers to the ability of a refugee household to manage and cope with the impact of the emergency or crisis [32].”Line 105, Page 6.

In addition, the term vulnerability and how it is further portrayed in our context is further described in the discussion section (pages 32 and 33).

- “Refugees, in Lebanon as with other humanitarian settings, are categorized based on legal status, registration and vulnerability, with each label carrying a significant weight. Vulnerability entails much meaning in the lives of refugees, as it is the main stratification category used by UNHCR and many other organizations to prioritize assistance allocation [69, 70]. The level of vulnerability is determined based on a number of set characteristics, which include household demographics, dependency, shelter conditions, and income [69, 70]. However, a question can arise as to what exactly characterizes a vulnerable refugee in Lebanon and other protracted conflicts when the majority of displaced individuals are living below the poverty line and are utilizing negative coping mechanisms of varying levels. Others argue that vulnerability as a criterion for assistance allocation and interventions is problematic due to its inherent exclusion of individuals from assistance [70]. In Lebanon, the sample studied in major assessments conducted by humanitarian agencies, including VASyR, are primarily composed of registered and recorded refugees [69]. Arguably, the most vulnerable population of unregistered refugees is left out of these assessments and subsequently humanitarian aid [69].” Line 651, Page 32.

References:

- Elina Virokannas, Suvi Liuski & Marjo Kuronen (2018): The contested concept of vulnerability: a literature review, European Journal of Social Work, DOI:

<https://doi.org/10.1080/13691457.2018.1508001>.

- World Food Programme (1998). Comprehensive Food Security & Vulnerability Analysis Guidelines. <https://documents.wfp.org/stellent/groups/public/documents/manual_guide_proced/wfp203208.pdf>

-DFID’s Sustainable Livelihoods Approach and its Framework: <http://www.glopp.ch/B7/en/multimedia/B7_1_pdf2.pdf>

*18. Here again identifying camp v city refugees in terms of vulnerability assessment can be informative to the humanitarian system*

**Authors’ response:** Thank you for the suggestion. Understanding the difference between urban and rural refugees, in addition to those residing in different types of housing is crucial. However, as the majority of vulnerable refugees reside in ITSs in the Beqaa region, the focus of our research was on this population. We hope that future research projects will be able to study the needs, vulnerabilities and coping mechanisms of urban refugees.

*19. Line 624: if you haven’t introduced the meaning of protracted displacement you might want to elaborate and also cite the sources.*

**Authors’ response:** Thank you for the suggestion. A definition of protracted crisis has been added.

- “However, the Syrian conflict and resulting refugee crisis can no longer be treated as acute, and require alternative measures contextualized for survival and more dignified livelihoods in protracted crises. In a protracted crisis, the life and/or livelihood of a great number of individuals is in danger for an extended period of time, with official institutions unable to secure their needs[72].”Line 677, Page 33.

*20. The definition of empowerment has proven to be controversial (especially in non-western contexts) so a definition and citation could be helpful here.*

**Authors’ response:** We appreciate the question, and we agree with the reviewer that the term empowerment that has been included can have different meanings and it was not defined properly in the statement on paradigm shifts needed. In fact, as we revised this sentence, we realized that the term empowerment was incorrectly included, as rather the focus of the statement and the referred to citation was on the lack of protection and security that refugees face in protracted and complex conflicts such as the case with the Syrian refugee crisis in host countries like Lebanon. The need for policies and systems that are more inclusive of refugees and displaced individuals is what was being referred to in that section. Thus, a minor revision was done as such:

- *“*A paradigm shift that takes into consideration several factors, including the protection of individuals and their human security, placing it at the forefront of all humanitarian programs and interventions rather than focusing on traditional state-centric ideas of security and on acute crisis management approaches [74].” Line 682, Page 33.

*21. Line 635: Other than a suggestion to integration into workforce, it is not clear what this study is suggesting as an alternative to the current humanitarian system and policies.*

**Authors’ response:** Thank you. We understand that the paradigm shift called for in the present study requires changes on multiple levels that can affect the humanitarian system in complex crises, as the case with the refugees in Lebanon. Authors attempted to propose several policy and programmatic-level interventions for consideration in the section “Implications for humanitarian systems and potential recommendations.” Please note that the integration of refugees into the workforce was one of these examples that was highlighted to provide them with a form of sustainable livelihood. A related recommendation was to informal health care (of Syrian nationality) with temporary registration to be able to provide increased access to health care services to their refugee communities, especially in remote areas. Also, authors proposed the need to examine complimentary interventions to food and cash transfer programs that can help improve food security of refugees while also contributing to other positive health outcomes (please see pages 34 and 35).

*22. Good summary, however, the contribution of the study and how it benefits different audience, including policy makers, humanitarian organizations, and researcher might need to be spelled out more clearly.*

**Authors’ response:** The reviewer’s comment is well-received. We reviewed the discussion section all throughout and the conclusion section to further highlight the novelty of the study findings and the contribution of this work to the literature as well as potential implications for health professionals and refugee health workers and researchers.

Sincerely,

Corresponding authors (on behalf of all co-authors)
